# Regional Transit Authority Efforts to Support COA Transportation in Rural Areas in Massachusetts

**DOI:** 10.1093/geroni/igaf122.1292

**Published:** 2025-12-31

**Authors:** Taylor Jansen, Shayna Gleason, Nina Silverstein, Caitlin Coyle, Scott Rich

**Affiliations:** University of Massachusetts Boston, Boston, Massachusetts, United States; International Transportation Learning Center / Transit Workforce Center, Washington, District of Columbia, United States; University of Massachusetts Boston, Boston, Massachusetts, United States; University of Massachusetts Boston, Boston, Massachusetts, United States; Montachusett Regional Transit Authority, Fitchburg, Massachusetts, United States

## Abstract

The Montachusett Regional Transit Authority (MART) serves both urban and rural areas within its 624-square-mile region, encompassing parts of Northern Worcester and Western Middlesex Counties in Massachusetts. MART, in cooperation with Councils on Aging (COA) in their region, sought to improve transportation services for older adults and people with disabilities and enable them to access a greater array of destinations, through the deployment of software for trip booking and management. Trip data were analyzed for N = 10 communities in June and September 2024 after the new software was introduced to COAs to assist with dispatching and reporting requirements. In addition, zoom interviews were conducted with N = 9 dispatchers, N = 6 drivers, N = 5 COA directors, and phone interviews with N = 21 older adult riders. An in-person focus group of riders was also held. Key findings included that dispatchers and directors appreciated being able to link rider information automatically, having voice call and text reminders to riders, and moving from “paper and pen” to more efficient reporting, though some software challenges remained. Overall, trip data revealed a slight increase in life-sustaining trips from June to September, while riders voiced an interest in also having more social and recreational destinations or “life-enriching” trips provided. MART’s interest in increased coordination across the towns remains a challenge while COAs have varying operations and town policies for travel across jurisdictions. Recommendations for the future include more robust training on the software and uses of its data for drivers and dispatchers, as well as incentivizing and enabling trip coordination between COAs.

